# Advances in Nanoparticles as Vaccine Adjuvants

**DOI:** 10.3390/vaccines14020159

**Published:** 2026-02-08

**Authors:** Sohrab Ahmadivand, Eduardo Gomez-Casado

**Affiliations:** 1 Faculty of Veterinary Medicine, Ludwig-Maximilians University Munich, 80539 Munich, Germany; sohrab.ahmadivand@lmu.de; 2 Department of Biotechnology, Instituto Nacional de Investigación y Tecnología Agraria y Alimentaria, Consejo Superior de Investigaciones Científicas (INIA-CSIC), 28040 Madrid, Spain

## 1. Introduction

The development of safe, effective, and durable vaccines remains a central goal in preventing infectious diseases in humans and animals. Modern non-replicating platforms offer advantages in safety, antigen specificity, and manufacturing scalability; however, they often exhibit limited intrinsic immunogenicity. As a result, the identification and optimization of effective adjuvants have become critical determinants of vaccine performance [1].

Adjuvants enhance immune responses primarily by activating innate pathways, including pattern recognition receptors (PRRs), and can also promote germinal center formation, T follicular helper (Tfh) cell differentiation, and the magnitude, quality, and durability of adaptive immunity, including Th1/Th2 polarization and antibody titers [2].

Classical adjuvants, including aluminum salts and oil-based emulsions (e.g., MF59^®^, Montanide), are widely used and generally effective but may have limited capacity to elicit robust cellular or mucosal immunity. Aluminum salts primarily induce antibody (Th2) responses [3], whereas oil-emulsion adjuvants can cause side effects, including local reactogenicity and, occasionally, systemic reactions [4]. Furthermore, they provide limited flexibility to fine-tune immune responses and may be suboptimal for vaccines targeting intracellular pathogens, rapidly mutating viruses, or complex antigens, highlighting the need for next-generation adjuvant systems with greater design precision.

Recent advances in nanotechnology have significantly enhanced vaccine adjuvant design by enabling precise control over antigen delivery and immune activation. Nanoparticles can be engineered with defined size, shape, surface chemistry, and degradation kinetics, which influence lymphatic trafficking, antigen uptake, and immune recognition [5]. These properties allow nanoparticles to act as active immunostimulatory platforms rather than passive carriers, integrating targeted antigen presentation, innate immune engagement, and controlled antigen release within a single construct. Such attributes drive enhanced humoral and cellular responses compared with traditional adjuvants and are a foundational rationale for next-generation vaccine design [6].

For instance, inorganic nanoparticles, including nano-sized aluminum salts, silica, and clay, improve upon conventional adjuvants by enhancing cellular uptake and activating innate immune pathways. Lipid-based nanoparticles and oil-in-water emulsions (e.g., MF59^®^, AS03^®^) further augment immune responses while maintaining favorable safety profiles, and their conversion to well-defined nanoscale formulations may enhance antigen delivery and immunogenicity [7]. Organic systems, such as chitosan nanoparticles, offer increased stability and immunogenicity. Lipid nanoparticles (LNPs) possess intrinsic adjuvant activity, activating innate immunity and promoting robust antibody responses. Self-assembling protein nanocages (SAPNs), such as ferritin, mimic viral size and structure, enabling multivalent antigen display, improved stability, and enhanced immunogenicity. Protein-based adjuvants like flagellin, which engage TLR5 [8], can be incorporated into SAPNs to further boost adaptive immunity and allow co-display and co-delivery with antigens [9].

This Special Issue, “Advances in Nanoparticles as Vaccine Adjuvants”, compiles original research and comprehensive reviews that collectively delineate the scope, mechanistic diversity, and translational relevance of nanoparticle-based adjuvant systems. The studies presented underscore that rationally designed nanoparticles can integrate structural organization, targeted delivery, and immune modulation to enhance vaccine efficacy, safety, and translational potential. Together, they establish nanoparticle-based adjuvants as a cornerstone of next-generation vaccine design for infectious diseases.

## 2. An Overview of Advances in Nanoparticles as Vaccine Adjuvants

A substantial body of work in this Special Issue highlights self-assembling protein nanocages (SAPNs) with pathogen-mimicking architectures as advanced vaccine platforms that enable multivalent antigen display and co-delivery of adjuvants. Bacterial self-assembling protein nanocages exploit nanoscale symmetry and repetitive antigen display to promote strong B-cell receptor crosslinking and effective adaptive immune responses, thereby enhancing the stability and immunogenicity of subunit vaccines, as reviewed by Lamontagne et al. [10].

SAPNs such as ferritin represent versatile, multifunctional platforms with broad applicability and have been shown to enhance antigen persistence, facilitate efficient uptake by antigen-presenting cells (APCs), and amplify immune activation in subunit, DNA, and mRNA vaccines. As noted by Ahmadivand [9], beyond structural antigen display and RNAi therapeutics, SAPNs can also co-deliver antigens and adjuvants. They achieve this either through multivalent surface display or internal encapsulation of protein or small-molecule adjuvants, promoting efficient APC activation, sustained immune stimulation, and reduced off-target toxicity. Flexible design strategies allow single or dual adjuvant configurations and can tailor Th1/Th2 immune bias. This SAPN-adjuvant approach offers controlled and modular immune modulation, improving vaccine safety and efficacy, particularly for viral infections.

Protein nanoparticles also support multivalent vaccine designs in which structurally distinct antigens can be presented simultaneously on a single scaffold. For example, Xia et al. [11]. developed a chimeric viral nanoparticle, P24-αTSR, that self-assembles into a 24-meric octahedral structure displaying both the norovirus protruding (P) domain and the Plasmodium αTSR domain. This dual presentation retains the functional conformations of both antigens and elicits strong pathogen-specific antibody responses in mice, demonstrating the potential of SAPNs to co-deliver multiple antigens while promoting potent adaptive immunity.

Structural stability is an important factor in enhancing adjuvant performance. In this context, Wong et al. [12]. developed thermally stable protein nanoparticles that maintain immunogenicity under elevated temperatures and induce durable humoral immune responses after a single immunization, suggesting that similarly stable SAPN platforms could achieve comparable benefits.

Polymeric nanoparticles are a well-established and versatile class of vaccine adjuvants, with immunological outcomes determined by material composition, degradation kinetics, and mechanical properties. As demonstrated by Cui et al. [13], biodegradable PLGA–PEG 25% nanoparticles exhibit antigen-dependent adjuvanticity: nanovaccines conjugated with the S. aureus exotoxin rHlaH35L elicited more robust humoral, cellular, and innate immune responses, as well as superior protective efficacy, compared with those carrying the cell-wall protein rSpam, or both antigens combined. This underscores the critical importance of rational antigen–nanoparticle pairing in optimizing vaccine potency. The influence of physical nanoparticle properties was further shown by Song et al. [14], who reported that nanoparticle stiffness and degradation kinetics regulate antigen accessibility and cellular uptake in a route-dependent manner in an S. aureus mouse model, resulting in enhanced humoral and cellular immunity compared with conventional alum adjuvants. In addition, Su et al. [15] demonstrated that encapsulation of ginseng stem-leaf saponins within PLGA nanoparticles improves adjuvant stability, promotes dendritic cell-mediated antigen presentation, and amplifies both mucosal and systemic immune responses while maintaining a favorable safety profile. Overall, these findings illustrate that antigen selection, nanoparticle physicochemical properties, and bioactive payloads directly govern the magnitude, quality, and durability of vaccine-induced immune responses, providing a rational framework for next-generation polymeric adjuvants.

Nanoparticles also play a pivotal role in the adjuvantation of nucleic acid and mucosal vaccines, settings in which effective immune stimulation is particularly challenging. Sousa de Pinho et al. [16] showed that yeast-derived β-glucan capsules function as biologically derived nanoparticles that enhance DNA vaccine uptake by macrophages and APCs, improving immune priming while maintaining minimal cytotoxicity. In mucosal vaccination, polymeric caffeic acid acts primarily as a nanoparticle-based antigen delivery system rather than a classical immune stimulant, promoting targeted transport to mucosal dendritic cells and eliciting both local and systemic immune responses [17]. Chitosan-based nanoparticles further illustrate this dual adjuvant–carrier functionality: their mucoadhesive properties, intrinsic immunostimulatory activity, and sustained antigen-release capacity make them highly effective for mucosal immunization [18]. Consistent with these findings, Yang et al. [19] demonstrated that surface-modified liposomes incorporating lipidated chitosan derivatives, such as oleoyl-quaternized chitosan (OTMC), function as self-adjuvanting intranasal vaccine platforms, eliciting robust systemic and mucosal immune responses and promoting functional antibody and cytokine production.

Lipid nanoparticles (LNPs) carrying mRNA have also been shown to actively stimulate innate immune pathways in addition to delivering their cargo, shaping downstream adaptive responses. However, formulation features such as PEGylation, while improving particle stability, can trigger complement activation and hypersensitivity reactions, highlighting the need for careful optimization to balance safety and immunostimulatory efficacy [20].

Several review articles place nanoparticle adjuvants within broader translational and disease-specific contexts. Comprehensive overviews of nanovaccine platforms demonstrate how nanoparticles enhance antigen protection, immune targeting, and response durability across bacterial and viral infections [21]. Beyond immunological effects, the clinical translation of nanoparticle adjuvants also requires consideration of manufacturability, scalability, and regulatory compliance, as systematically analyzed by Filipić et al. [7]. Disease-specific applications further illustrate the value of these platforms. In tuberculosis vaccination, Wang et al. [22] emphasized that nanoparticle adjuvants improve antigen presentation kinetics and promote sustained cellular immunity, which is essential for protection against intracellular pathogens. Similarly, in aquaculture, nanoparticle-based formulations offer welfare-conscious alternatives to traditional oil- or aluminum-based adjuvants, enhancing vaccine efficacy while minimizing adverse effects in teleost fish [23].

Innovative strategies expanding the functional capabilities of nanoparticle adjuvants have been demonstrated through controlled antigen release systems. Brubaker et al. [24] showed that antigen-containing powders coated via atomic layer deposition enabled tunable delayed-release kinetics, providing precise temporal control over immune stimulation and supporting the development of single-administration vaccination strategies. Complementing this approach, Shalash et al. [25] highlighted that nanoparticle-compatible formulations can enhance peptide-based vaccines, which often suffer from low intrinsic immunogenicity. Optimized peptide antigens, particularly when conjugated to T-helper epitopes such as PADRE, elicited robust and functional antibody responses, including strong opsonic activity, even under complex co-infection conditions, reinforcing the role of nanoparticles in overcoming fundamental limitations of peptide vaccines.

## 3. Conclusions

This Special Issue demonstrates that nanoparticles function as active, designable vaccine adjuvants rather than passive carriers. Platforms including self-assembling protein nanocages, polymeric systems, lipid-based carriers, and nucleic acid formulations enhance antigen stability, enable multivalent and targeted presentation, and overcome intrinsic limitations of subunit, peptide, mucosal, and nucleic acid vaccines. Rational design, considering antigen selection, structural stability, physicochemical properties, and co-delivered adjuvants, directly governs the magnitude, quality, and durability of immune responses. Collectively, these studies underscore the potential of nanoparticle-based platforms to advance next-generation vaccines for both human and veterinary applications, with future efforts focusing on precision engineering, controlled or single-dose delivery, mucosal targeting, and integration with emerging technologies to develop safe, scalable, and highly protective vaccines.
